# P-1333. Influence of Allocation Proportion of Intervention and Control on Results Analyzed Using the Cox Proportional Hazard Model: A Pilot Meta-Analysis Using Actual Clinical Trials

**DOI:** 10.1093/ofid/ofae631.1511

**Published:** 2025-01-29

**Authors:** Soichi Takeishi, Tatsuo Inoue, Koichi Miyamura

**Affiliations:** Inuyama Chuo General Hospital, Inuyama-city, Aichi, Japan; Inuyama Chuo General Hospital, Inuyama-city, Aichi, Japan; Inuyama Chuo General Hospital, Inuyama-city, Aichi, Japan

## Abstract

**Background:**

In reports assessing the effect of an intervention (I) on COVID-19–related endpoints using the Cox proportional hazard model, “allocation proportions of I and control (C)” vary. The effect of these allocation proportion on results analyzed using Cox regression in actual clinical trials is unknown. Therefore, we conducted a pilot meta-analysis of clinical trials to investigate this effect.

The correlations between metrics

**Figure:**
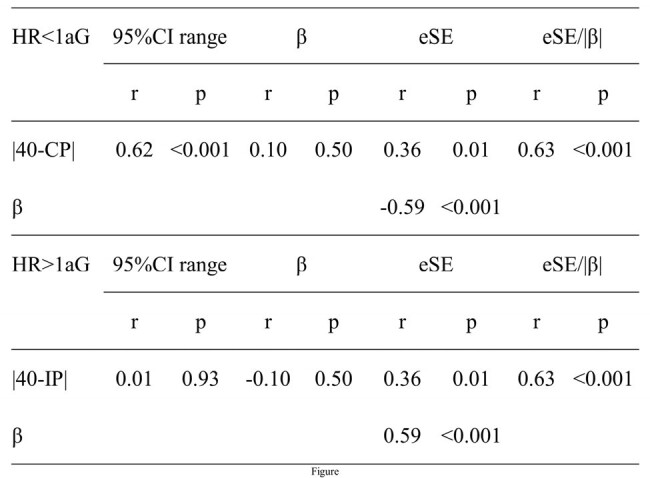

**Methods:**

Articles published in The New England Journal of Medicine were included as part of a pilot meta-analysis if the following conditions were satisfied: 1. assessing endpoints related to COVID-19, and 2. using Cox regression. We referred to “upper limit of 95% confidence interval (CI) of hazard ratio (HR) (U95%CI) − lower limit of 95%CI of HR (L95%CI)” as the 95%CI range. Partial regression coefficients (β) were calculated as Log_e_(HR), and the standard error of β was estimated using ((Log_e_(U95%CI) – Log_e_(HR)) ÷ 1.96 + (Log_e_(HR) − Log_e_(L95%CI)) ÷ 1.96) ÷ 2, denoted as eSE. We calculated eSE ÷ an absolute value of β (eSE/|β|). The number of subjects (n) in an intervention group (nI) ÷ (nI + n in a control group (nC)) × 100 was termed “intervention proportion % (IP)”, and nC ÷ (nI + nC) × 100 was termed “control proportion % (CP)”. We calculated the absolute value of 40 − CP (|40 − CP|) and 40 – IP (|40 – IP|). For HR< 1, “HR< 1 adjusted group” (HR< 1aG) retained the original metrics, while the “HR >1 adjusted group” (HR >1aG) used metrics calculated with the reciprocal of HR and its 95%CI (e.g. 0.50 (0.20-0.80) → 2.00 (1.25-5.00)). For HR >1, HR< 1aG used metrics calculated with the reciprocal of HR and its 95%CI, while HR >1aG retained the original metrics.

**Results:**

We included 50 outcomes from 22 studies. The figure shows the correlations between metrics. In HR< 1aG, |40 − CP| correlated with the 95%CI range (r=0.62, p< 0.001), whereas |40 – IP| did not correlate with the 95% CI range (r=0.01, p=0.93) in HR >1aG. In HR< 1aG, |40 − CP| correlated with the eSE/|β|, and |40 − IP| correlated with the eSE/|β| in HR >1aG (both r=0.63, p< 0.001).

**Conclusion:**

The present study results may indicate that extremely biased allocation proportions of I and C diminish the credibility of HR. Replacing eSE/|β| with 95%CI range may not accurately assess this diminished credibility.

**Disclosures:**

**All Authors**: No reported disclosures

